# A comprehensive dataset of damaged banknotes in Indian currency (Rupees) for analysis and classification

**DOI:** 10.1016/j.dib.2023.109699

**Published:** 2023-10-18

**Authors:** Vidula Meshram, Vishal Meshram, Kailas Patil, Yogesh Suryawanshi, Prawit Chumchu

**Affiliations:** a Vishwakarma University, Pune, PIN-411038, India; b Vishwakarma Institute of Information Technology, Pune, PIN-411038, India; c Kasetsart University, Sriracha, Thailand

**Keywords:** Classification, Currency recognition, Damaged banknotes, Indian currency, Spoilt banknotes

## Abstract

Detecting authentic and quality banknotes presents a significant challenge, particularly for individuals with low vision or visual impairments. Extensive research has been dedicated to achieving accurate banknote detection. It is crucial for clean banknotes to be readily detectable and accepted in daily transactions. However, existing Indian currency datasets suffer from limitations, including insufficient size, a lack of datasets on damaged/spoiled banknotes, and the unavailability of publicly accessible datasets featuring spoiled, torn, or altered banknotes. Recognizing the vital importance of a spoiled banknote dataset for the benefit of low vision and visually impaired individuals, we introduce a comprehensive dataset of spoiled banknotes comprising 5125 Indian currency notes. This dataset encompasses both old and new denominations of 10, 20, 50, and 100 Rupees, aiming to significantly enhance the accessibility and accuracy of banknote detection systems. By making this dataset openly accessible to the researchers, we aim to promote research and development of solutions for detection of spoiled banknote.

Specifications Table

**Table:** 

Subject	Applied Machine Learning
Specific subject area	Currency, Classification and Recognition
Type of data	Images of Spoilt banknotes of Indian currency (Rupees)
How data were acquired	The acquisition of the Indian banknote dataset involved capturing images using a high-resolution camera on a mobile phone.
Data format	Raw
Description of data collection	The dataset encompasses resized images of damaged Indian banknotes. The images were captured using a high-resolution mobile phone camera and saved in the original .jpg format with dimensions of 3024 × 4032 pixels. To standardize the dataset, all images were subsequently resized to a dimension of 1280 × 768 pixels. Within the dataset, there are eight classes representing different denominations of Indian banknotes: New 10 Rupees, Old 10 Rupees, New 20 Rupees, Old 20 Rupees, New 50 Rupees, Old 50 Rupees, New 10 Rupees, and Old 100 Rupees. The currency notes images were captured under diverse environmental conditions, encompassing scenarios such as dark backgrounds, illuminated backgrounds, cluttered environments, and from different perspectives, including both front and back angles. The dataset includes banknotes that exhibit different forms of damage, such as being soiled, slightly or completely torn, mutated, or crumbled, ensuring a comprehensive representation of damaged Indian banknotes.The dataset contains the resized images, providing researchers with flexibility and options for their specific needs and analysis.
Data source location	VISHWAKARMA UNIVERSITY, Pune Laxmi Nagar, Kondhwa Budruk, Pune - 411 048. Maharashtra, India. Latitude and longitude: 18.4603°N, 73.8836°E
Data accessibility	Repository name: Dataset of Spoilt Banknotes of India (Rupees) Data identification number: 10.17632/jh6979fg2t.4 Direct URL to data: https://data.mendeley.com/datasets/jh6979fg2t/4

## 1 Value of the Data

• *Comprehensive and Diverse*: The dataset comprises 5125 high-quality images, encompassing eight different classes of spoilt Indian banknotes. It includes both old and new denominations, providing a comprehensive representation of spoilt banknotes in Indian currency.
• *First Open-Access Dataset*: To the best of our knowledge, this dataset is the first openly accessible collection of spoilt Indian banknotes. Its availability enables researchers and practitioners to access and utilize the dataset for various applications and research purposes.
• *Identification and Classification*: The dataset serves as a valuable resource for researchers developing algorithms and models to identify and classify spoilt and non-spoilt Indian banknotes accurately. It facilitates advancements in the field of banknote analysis and classification.
• *Accessibility for Visually Impaired*: The dataset can contribute to the development of tailored applications that aid visually impaired individuals in identifying non-spoilt banknotes. This application can enhance their independence and ease of conducting financial transactions.
• *Sorting Applications*: The dataset holds significance for building applications that can effectively sort spoilt and non-spoilt banknotes. This has practical implications for banks, government agencies, and other practitioners involved in monetary transactions, streamlining their processes, and improving efficiency.

## 2 Objective

The objective of this study is to develop a dataset and associated methodologies for the accurate classification of spoilt and non-spoilt banknotes in the context of day-to-day monetary transactions. It is essential to correctly differentiate between clean and unspoilt banknotes, which are widely accepted by local vendors, and spoilt banknotes, including torn, holed, crumbled, or soiled notes. Such spoilt banknotes can only be exchanged at designated public sector banks, following the guidelines set by the Reserve Bank of India [6].

Currency recognition and automated teller machines play a vital role in correctly identifying banknotes [7]. However, visually impaired individuals face significant challenges, including currency recognition, during their financial transactions [8,9]. Therefore, there is a need to develop a system that can verify the authenticity of banknotes [[1], [10]].

While several currency datasets are currently available, there is a scarcity of specific datasets focusing on spoilt banknotes for experimentation by researchers [[4], [5], [11]]. To address this gap, the primary objective of this study is to create a comprehensive dataset of spoilt banknotes. This dataset will facilitate the development and evaluation of algorithms and systems capable of sorting damaged and undamaged banknotes accurately.

## 3 Data Description

The dataset used in this study builds upon the existing dataset of Indian (Rupee) and Thai (Bhatt) banknotes with annotations, created by [2,12], which has been widely utilized by researchers for tasks such as recognition and classification of Indian and Thai banknotes. In this work, we extend this dataset by creating a new dataset specifically focused on spoilt Indian banknotes.

The spoilt banknotes dataset comprises eight distinct classes, namely: New 10 Rupees, Old 10 Rupees, New 20 Rupees, Old 20 Rupees, New 50 Rupees, Old 50 Rupees, New 10 Rupees, and Old 100 Rupees. These denominations were selected as they are commonly used in day-to-day transactions by the general public.

To create this dataset we collected the spoiled banknotes from vegetable vendors, local shopkeepers, and neighbors. We followed the Reserve Bank of India (RBI) guidelines to authenticate Indian banknotes. As per the RBI guidelines we examined each spoiled banknote in the dataset by scrutinizing it for key security features such as watermarks, security threads, and intaglio printing. Additionally, we verify that the year of printing, the language panel, and the theme on the reverse side align with the designated denomination [13,14]. To ensure the dataset's diversity and applicability, the images of spoilt banknotes were captured in various environments and backgrounds. This includes illuminated and dark settings, cluttered backgrounds, as well as capturing the banknotes from the front and back directions. The dataset encompasses images of slightly and completely torn, soiled, and crumbled banknotes, reflecting real-world scenarios.

**Fig. 1:**
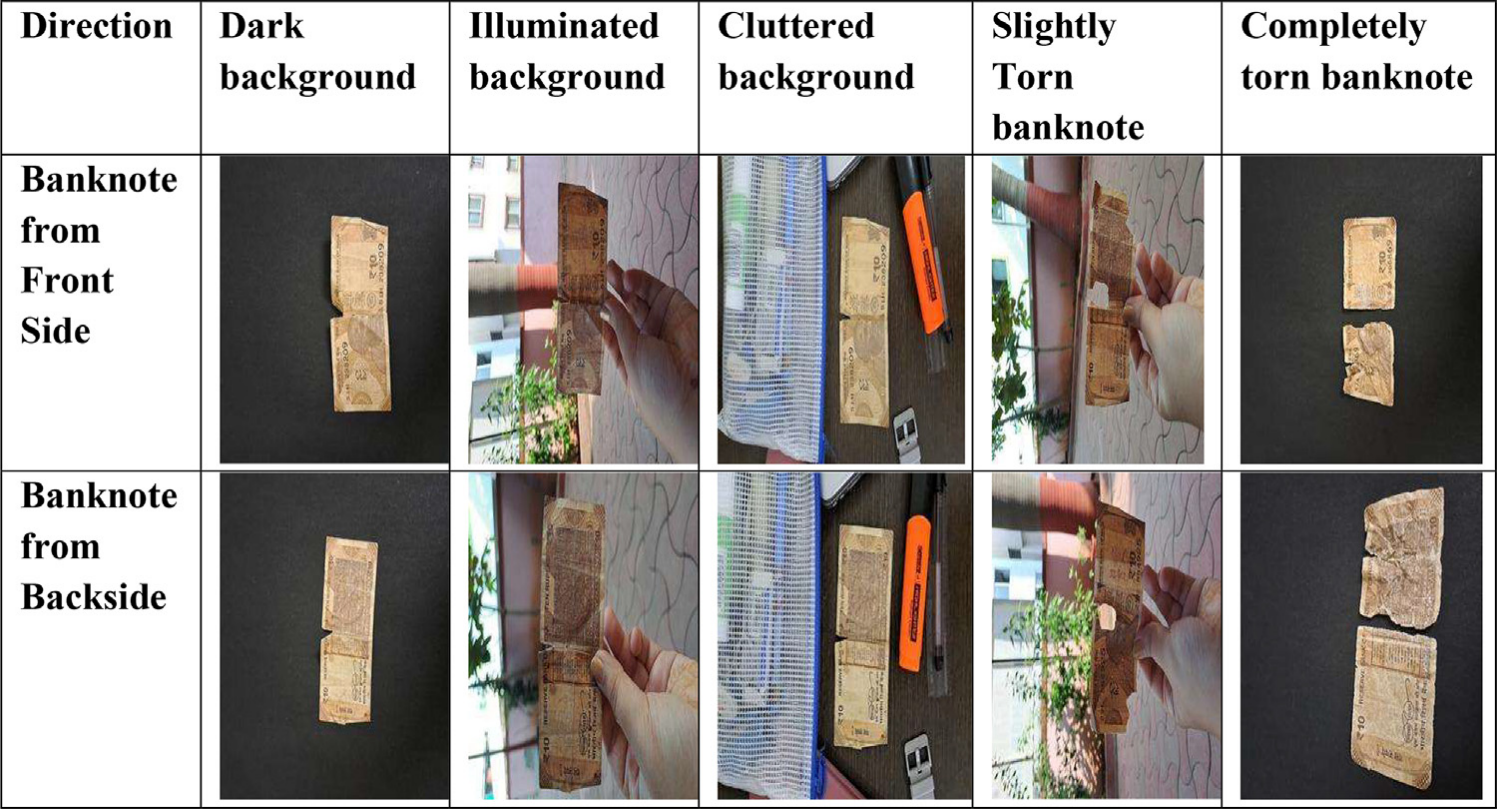
Banknote images captured in diverse environments.

The dataset is organized and stored in specific folders according to the respective denominations. Fig. 1 showcases a sample of the images contained within the dataset, exemplifying the variations in environments and conditions.

**Fig. 2:**
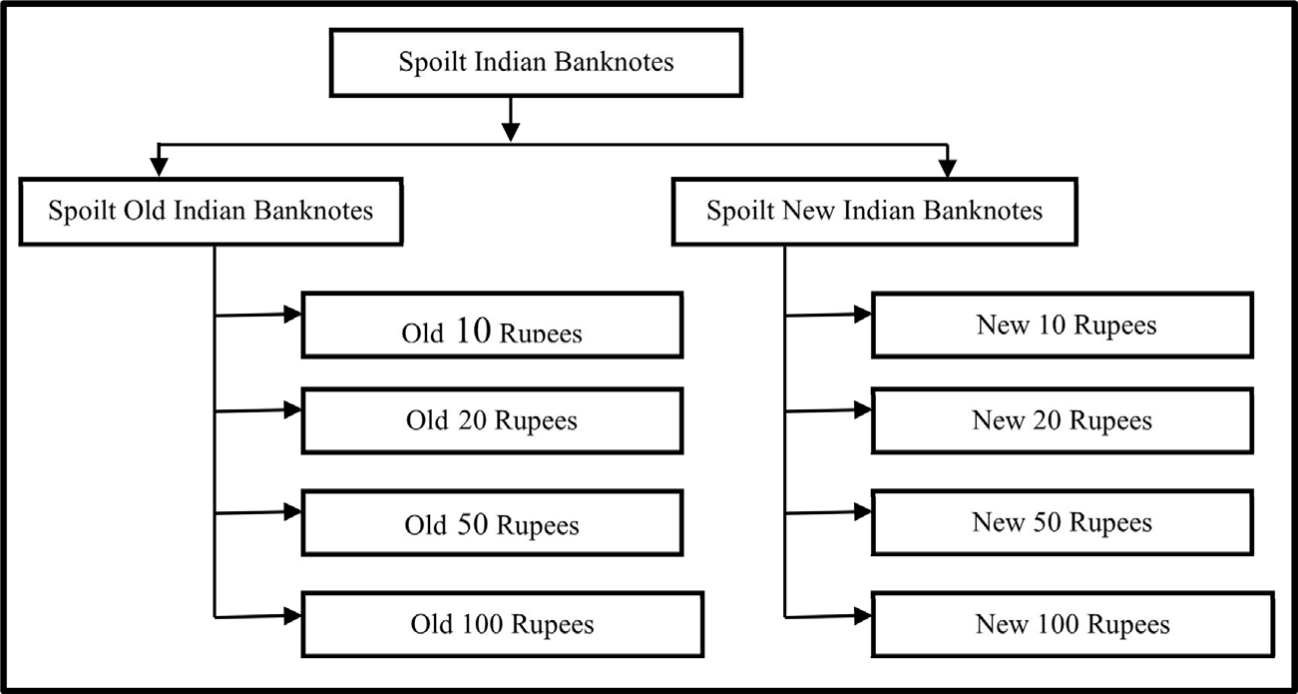
Directory structure of the spoilt banknote dataset.

The dataset of spoilt banknotes comprises images that were collected in diverse environments and backgrounds, including illuminated, dark, cluttered, and from both the front and back directions of the banknotes [3]. The images capture spoilt banknotes that range from slightly torn, soiled, to completely crumbled notes, thus encompassing a variety of conditions commonly encountered in real-world scenarios. To ensure organization and ease of use, the dataset's images are stored in separate folders based on the denomination of the banknotes. This directory structure allows for convenient access and management of the dataset. You can refer to Fig. 2 for a visual representation of the banknote image dataset directory structure.

## 4 Experimental Design, Materials, and Methods

### 4.1 Experimental design

The spoilt banknote dataset was generated through the acquisition of images using the high-resolution rear cameras of a Samsung A33 5G mobile and an Apple iPhone 6. The mobile cameras were employed to capture images of spoilt banknotes from both the old and new categories.

To standardize the dataset, the original images underwent preprocessing, which involved resizing them to a dimension of 1280 × 768 pixels. Following the resizing process, the images were organized and saved in separate folders based on their respective denominations. This organizational structure allows for convenient access and management of the dataset.

The steps involved in the spoilt banknote image data acquisition process are outlined in Table 1. The images of spoilt banknotes from both the old and new categories were captured during daytime and evening hours within the months of October 2022 to November 2022 and May 2023 to June 2023. Subsequently, the captured images were resized and carefully organized, ensuring that they were properly segregated and saved in respective folders based on their corresponding denominations.

**Table 1:** Data acquisition steps.

Sr. No.	Step	Duration	Activity
1.	Image Acquisition	October to November 2022 and May to June 2023	Daily during daytime for illuminated environment and at evening for dark environment the spoilt banknote images were captured.
2.	Image Pre-processing	December 2022 and June 2023	The images appropriate for dataset were selected from gathered images and were pre-processed.

### 4.2 Materials or specification of image acquisition system

The cameras used in the data acquisition process and the specifications of the captured images:

1. For Samsung Galaxy A33 5G Android Mobile:
  • Make and Model: Samsung Galaxy A33 5G Android Mobile.
  • Rear Primary Camera: It has a 48-megapixel (f/1.8) lens.
  • Camera Sensor: The camera sensor used is Sony IMX 582 1/2".
  • Battery: The mobile is equipped with a 5000 mAh battery.
2. For Apple iPhone 6:
  • Make and Model: Apple iPhone 6.
  • Rear Camera: It has an 8-megapixel (f/2.2) lens with rear autofocus capability.
  • Battery: The iPhone 6 has a 1080 mAh battery.

In both cases, the captured images were saved in JPG format and resized with a resolution of 1280 × 768 pixels. These specifications provide essential information about the cameras and image properties utilized in the data acquisition process.

## 3.3 Method

Samsung Galaxy A33 5G Android Mobile with primary rear camera of 48 megapixel and Apple iphone6 rear camera is used to capture spoilt banknote images. These images are captured in different back grounds. The original images with size 3024 × 4032 and 2448 × 3264 were resized to 1280 × 768 pixel. Table 2 describes the classes, number of image taken and the environments in which images are taken.

**Table 2:** Banknote details.

Types of images gathered	Currency note images captured in varied backgrounds	Bank notes	Denominations considered	Count of images for each denomination	Total number of images
Front Direction , Backward Direction, Rotated, Soiled, Slightly torn, Completely torn	Illuminated, Dark, cluttered	Spoilt Old India Banknotes	Old 10 Rupees	650	**2584**
			Old 20 Rupees	647	
			Old 50 Rupees	633	
			Old 100 Rupees	654	

Front Direction, Front Direction Rotated, Soiled, Slightly torn, Completely torn	Illuminated, Dark, cluttered	Spoilt New India Banknotes	New 10 Rupees	645	**2541**
			New 20 Rupees	625	
			New 50 Rupees	632	
			New 100 Rupees	639	
**Total number of images in the dataset:**					**5125**

## Ethics Statement

The proposed data does not involve studies with animals, humans or data collected from social media platform. Therefore, we confirm that our research strictly adheres to the guidelines for authors provided by Data in in terms of ethical considerations.

## CRediT authorship contribution statement

**Vidula Meshram:** Methodology, Data curation, Writing – original draft. **Vishal Meshram:** Supervision. **Kailas Patil:** Conceptualization, Supervision, Writing – review & editing. **Yogesh Suryawanshi:** Writing – review & editing. **Prawit Chumchu:** Writing – review & editing.

## Data Availability

Dataset of Spoilt Banknotes of India (Rupees) (Original data) (Mendeley Data).
